# New perspective on single-radiator multiple-port antennas for adaptive beamforming applications

**DOI:** 10.1371/journal.pone.0186099

**Published:** 2017-10-12

**Authors:** Gangil Byun, Hosung Choo

**Affiliations:** 1 Metamaterial Electronic Device Research Center, Hongik University, Seoul, Korea; 2 School of Electronic and Electrical Engineering, Hongik University, Seoul, Korea; Beijing University of Posts and Telecommunications, CHINA

## Abstract

One of the most challenging problems in recent antenna engineering fields is to achieve highly reliable beamforming capabilities in an extremely restricted space of small handheld devices. In this paper, we introduce a new perspective on single-radiator multiple-port (SRMP) antenna to alter the traditional approach of multiple-antenna arrays for improving beamforming performances with reduced aperture sizes. The major contribution of this paper is to demonstrate the beamforming capability of the SRMP antenna for use as an extremely miniaturized front-end component in more sophisticated beamforming applications. To examine the beamforming capability, the radiation properties and the array factor of the SRMP antenna are theoretically formulated for electromagnetic characterization and are used as complex weights to form adaptive array patterns. Then, its fundamental performance limits are rigorously explored through enumerative studies by varying the dielectric constant of the substrate, and field tests are conducted using a beamforming hardware to confirm the feasibility. The results demonstrate that the new perspective of the SRMP antenna allows for improved beamforming performances with the ability of maintaining consistently smaller aperture sizes compared to the traditional multiple-antenna arrays.

## Introduction

The use of arrays with multiple antennas has become essential for adaptive beamforming in advanced wireless communication systems. Typically, arrays are used to adjust the direction of beams and nulls by multiplying complex weights to antenna ports for more reliable communication links in a multipath environment. This beamforming capability can be extended to estimate the direction of signals and mitigate the effects of interference; however, a growing demand for more effective beamforming capability in handheld devices with limited space has led to several technical challenges from an antenna engineering standpoint. First, active element patterns are distorted by strong mutual coupling between the array elements [1–9]. Second, these distorted radiation characteristics result in lower estimation accuracy, increased ambiguity, and poor resolution in adaptive beamforming operations [10–16]. Although various miniaturization techniques have been applied to the radiating elements in an effort to employ more antennas in a limited space [17–19], the antennas experience an additional gain reduction with an undesired frequency shift due to the narrow matching bandwidth, which cannot exceed the fundamental bandwidth limit [20]. Thus, there has been a growing demand for altering the traditional approach of multiple-antenna arrays to solve these technical challenges, which allows for improving the beamforming performance with a miniaturized aperture size.

In this paper, we propose an innovative approach to more effective beamforming operations using a novel single-radiator multiple-port (SRMP) antenna to alter the traditional multiple-antenna array in the case of an extremely restricted aperture area. The proposed SRMP antenna employs a single microstrip radiator printed on a dielectric substrate, and multiple ports are connected to the radiator to enable beamforming without enlarging the aperture. The traditional purpose of the multi-port geometry is to achieve either directive patterns or multiple-band resonances with a single radiator [21–22]; however, the major contribution of this paper is to provide a new perspective on beamforming capability using the SRMP antenna for use as the extremely miniaturized front-end component in adaptive beamforming applications. In our approach, the radiation characteristics for each port of the SRMP antenna are theoretically formulated using the cavity model [23], and the formulated fields are used to electromagnetically characterize the array factor for use as complex weights in various beamforming applications, such as the direction-of-arrival (DoA) estimation, interference mitigation, and multiple-input multiple-output (MIMO) antennas. To explore the fundamental performance limits of the SRMP antenna according to the miniaturization of the aperture size, the dielectric constant of the substrate is adjusted from 2 to 36, and variations of beamforming performance with respect to the aperture size are investigated and compared with the traditional multiple-antenna arrays, denoted as multiple-radiator multiple-port (MRMP) arrays. The results are then rigorously confirmed by full-wave electromagnetic (EM) simulations and measurements on active element patterns of three sample SRMP antennas that are fabricated on substrates with dielectric constants of 2.2, 4.5, and 10. The validity of the SRMP antenna is further verified through field tests using beamforming hardware composed of the universal software radio peripheral (USRP) [24], OctoClock [25], a power splitter [26], and an Ethernet switch [27]. The results prove that the beamforming capability of the SRMP antenna can be theoretically characterized and experimentally demonstrated. In addition, it is evident that the SRMP antenna is more appropriate than the traditional MRMP array for aperture miniaturization with improved beamforming performance, despite the extremely reduced aperture size.

## Physical implementation

### Electromagnetic characterization of single-radiator multiple-port antennas

**Fig 1:**
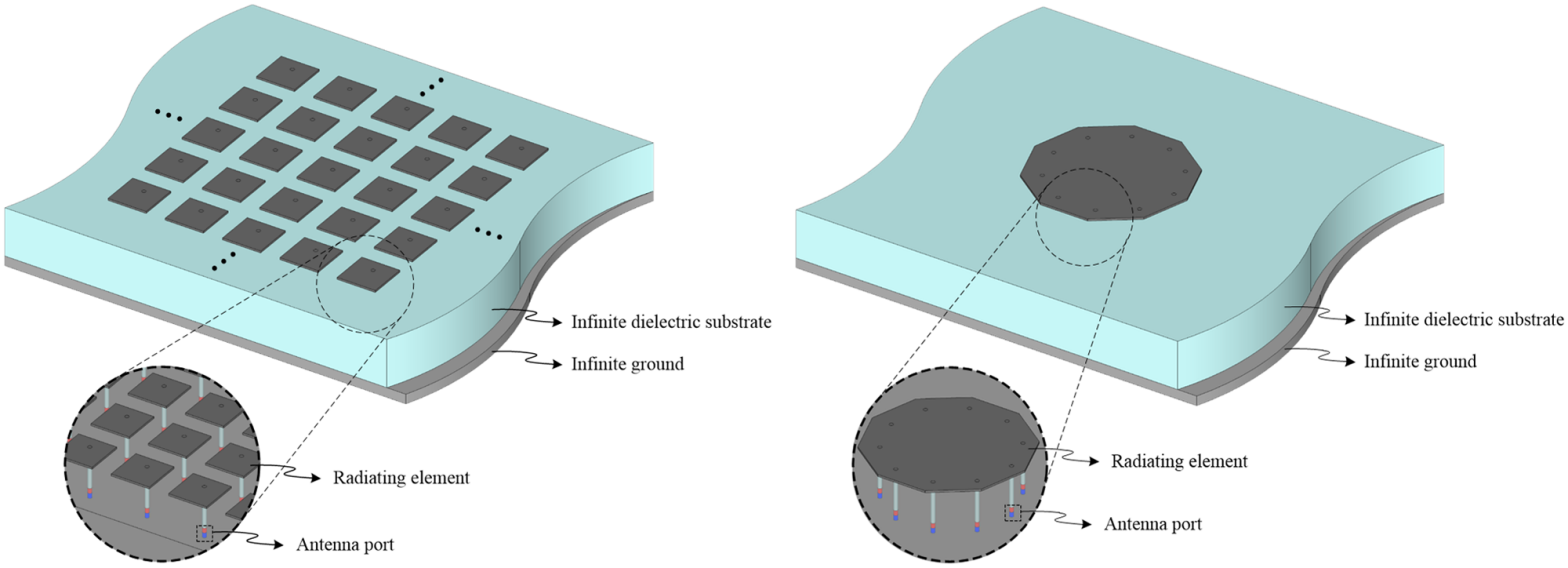
Conceptual geometries of the novel SRMP antenna and the traditional MRMP array. The geometry of the SRMP antenna with an *N*-sided polygon patch connected to multiple ports is conceptually compared to that of the multiple-antenna array, denoted as MRMP array. (A) Traditional MRMP array composed of *N* identical patch antennas. (B) Proposed SRMP antenna with *N* ports.

Fig 1 shows the conceptual geometry of an *N*-port SRMP antenna, which represents an alternative approach to the *N*-port MRMP array and is designed to improve beamforming performance with much smaller aperture sizes. Traditionally in the MRMP array, each antenna port is connected to a separate radiating element to form an adaptive array pattern by fully exciting the array with complex weights; however, the ports of the SRMP antenna feed a single *N*-sided polygon patch that is printed on a dielectric substrate with the ground. Note that the proposed SRMP antenna follows the typical design scheme for a stand-alone microstrip patch antenna; thus, the aperture size of the radiating patch is designed to be approximately half of an effective wavelength at the resonant frequency. This implies that the SRMP antenna occupies less space than the MRMP array; for example, the aperture area of a four-port MRMP array with a 2 × 2 is four times larger than that of a four-port SRMP antenna. Since the aperture area of the radiating patch is inversely proportional to the relative permittivity (*ε*_*r*_) of the substrate, the space occupancy of the SRMP antenna is consistently less than that of the MRMP array, regardless of *ε*_*r*_.

**Fig 2:**
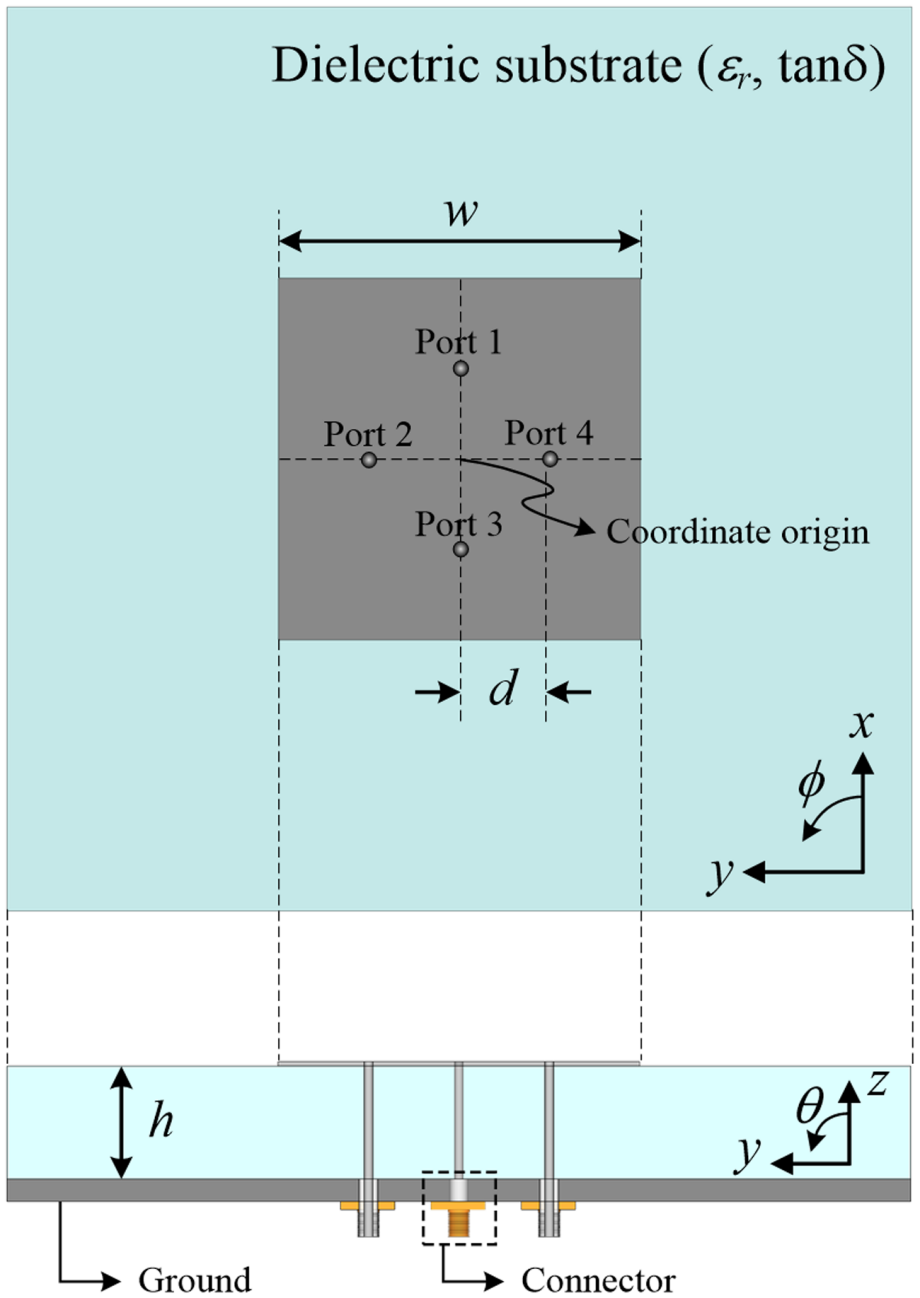
Four-port SRMP antenna employed in the derivation of the unique array factor *AF*_*SRMP*_. For ease of derivation, the number of ports is limited to four, which allows the radiating fields to be analytically formulated for a square patch with an edge length of *w*. The patch is printed on a dielectric substrate with thickness *h* and is fed by four coaxial probes at distance *d* from the center.

To provide the new perspective on the SRMP antenna, beamforming capability is then theoretically formulated with the assumption *N* = 4. Fig 2 illustrates that a square patch with edge length *w* is fed by four coaxial probes located at distance *d* from the center, and the patch is printed on a substrate with thickness *h*. The radiated field of the patch antenna can be formulated using the cavity model that replaces the electric field induced between the edge of the patch and the ground with an equivalent magnetic current density as an aperture source of the radiating slot mounted on an infinite plate [15]. Thus, the far-zone field of the SRMP antenna at the *n*-th port can be approximated by an array of two radiating slots, which can be expressed as follows:

$$E_{\theta }^{n}=-j\frac{k_{0}hwE_{0}e^{-jk_{0}r}}{2\pi r}\left\{\text{cos}\left(\phi -\phi_{n}\right)\left(\frac{\text{sin}Y_{n}}{Y_{n}}\right)\left(\frac{\text{sin}Z}{Z}\right)\right\}AF_{slot}^{n},$$ (1)

where *E*_*0*_ is a constant, and *r* is the distance from the origin to the observation point in the far zone. *k*_*0*_ is the propagation constant in free space, and *n* is an index of the antenna port. *ϕ*_*n*_ is an angular position of the *n*-th port measured from the *x*-axis, when the center of the patch is the origin of the rectangular coordinate. The substituted variables of *Y*_*n*_ and *Z* can be written as:

$$Y_{n}=\frac{k_{0}w}{2}\text{sin}\theta \text{sin}\left(\phi -\phi_{n}\right)$$ (2)

$$Z=\frac{k_{0}h}{2}\text{cos}\theta .$$ (3)

$AF_{slot}^{n}$ indicates the two-element array factor of the two radiating slots with an inter-element spacing of *w*, which is given by

$$AF_{slot}^{n}=2\text{cos}\left(X_{n}+\beta_{d}\right),$$ (4)

where

$$X_{n}=\frac{k_{0}w}{2}\text{sin}\theta \text{cos}\left(\phi -\phi_{n}\right).$$ (5)

*β*_*d*_ represents the phase difference between the two radiating slots as defined by

$$\beta_{d}=-\frac{k_{0}}{2}\frac{d}{\sqrt{\varepsilon_{reff}}}$$ (6)

and is a function of *d* and the effective dielectric constant *ε*_*reff*_, which is denoted as

$$\varepsilon_{reff}=\frac{\varepsilon_{r}+1}{2}+\frac{\varepsilon_{r}-1}{2}{\left[1+12\frac{h}{w}\right]}^{-\frac{1}{2}}.$$ (7)

In the cavity model, *β*_*d*_ is approximated to zero for a conventional rectangular patch antenna with a single port at the resonant frequency. However, the SRMP antenna has a non-zero value of *β*_*d*_ because the presence of other ports sharing the same radiator perturbs the phase of the equivalent magnetic current density.

**Fig 3:**
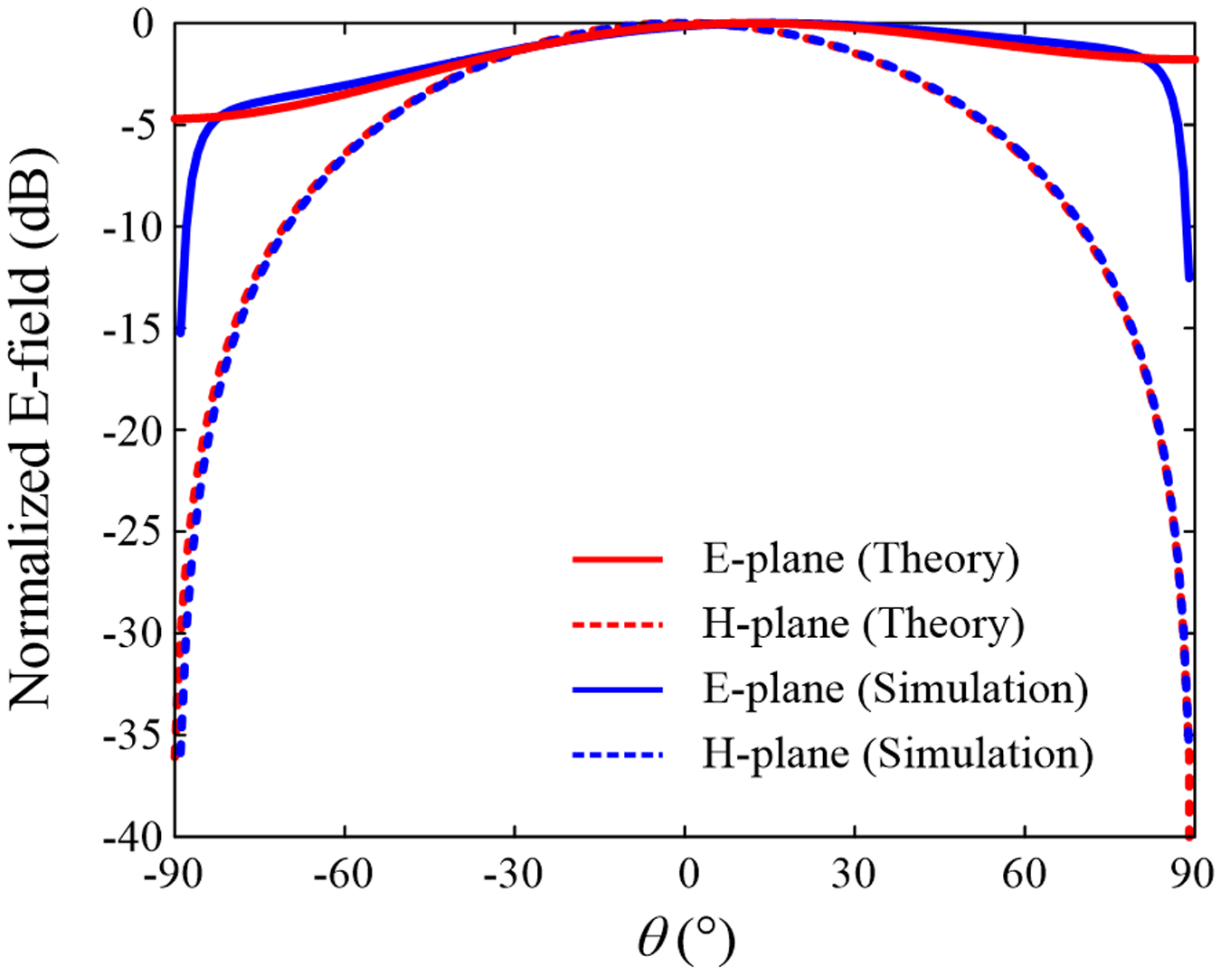
Comparison of far-zone fields between the derived equations and the full-wave EM simulation. Theoretically-driven electric fields in the E-plane (red solid line) and H-plane (red dotted line) are calculated using Eq (1) and are compared to the results of the full-wave EM simulation, as specified in blue. Due to the phase delay *β*_*d*_, the E-plane field is steered toward the positive *θ*-direction, and the deviation between the gain value of *θ* = +90° and that of *θ* = ‒90° is 2.9 dB.

Fig 3 presents a comparison of far-zone fields observed at Port 1, and the design parameters used in this comparison are *w* = 47.6 mm, *h* = 2.5 mm, *d* = 10 mm, and *ε*_*r*_ = 4. The red lines indicate the normalized electric fields calculated by Eq (1) in the E- and H-planes, and the blue lines represent the results obtained from the full-wave EM simulation^16^, which are provided to verify the reliability of the derived formulations. In our EM simulation, it is assumed that the rectangular patch is printed on an infinite substrate with an infinite ground covered by the perfect electric conductor. Thus, the field strengths at ±90° become zero with weak field strengths of –15.2 dB and –12.5 dB at –89.9° and +89.9°, respectively [23]. It is important to point out that the pattern of the E-plane is steered slightly toward the positive *θ*-direction, whereas that of the H-plane remains symmetrical (*see* S1 Fig in the supplementary information to verify the trend of the steered angle when *ε*_*r*_ = 2 and *ε*_*r*_ = 10). This is a unique feature of the SRMP antenna, which is determined by the factor of *β*_*d*_ in Eq (6), and it cannot be observed in conventional stand-alone microstrip patch antennas having a single port.

The steered pattern caused by the non-zero *β*_*d*_ allows each port of the SRMP antenna to provide a unique phase signature depending on the observation angle, and this phase signature is essential for the beamforming capability to define the array factor from the total field $E_{\theta }^{t}$, which can be expressed as

$$\begin{array}{l} E_{\theta }^{t}=E_{\theta }^{1}+E_{\theta }^{2}+\cdots +E_{\theta }^{N}\\ \;\;\;\;\;\;=-j\frac{k_{0}hwE_{0}e^{-jk_{0}r}}{\pi r}\left(\frac{\text{sin}Z}{Z}\right)\sum_{n=1}^{N}\left[\text{cos}\left(\phi -\phi_{n}\right)\left(\frac{\text{sin}Y_{n}}{Y_{n}}\right)\text{cos}\left(X_{n}+\beta_{d}\right)\right]. \end{array}$$ (8)

Thus, the unique definition of the array factor for the SRMP antenna is given by

$$AF_{SRMP}=\sum_{n=1}^{N}\left[\text{cos}\left(\phi -\phi_{n}\right)\left(\frac{\text{sin}Y_{n}}{Y_{n}}\right)\text{cos}\left\{\frac{k_{0}}{2}\left(w\text{sin}\theta \text{cos}\left(\phi -\phi_{n}\right)-\frac{d}{\sqrt{\varepsilon_{reff}}}\right)\right\}\right].$$ (9)

It is important to point out that *AF*_*SRMP*_ is distinguished from the array factor of the MRMP array, that is,

$$AF_{MRMP}=\sum_{n=1}^{N}e^{jk_{0}{\hat{a}}_{r}\cdot \bar{p}},$$ (10)

where

$${\hat{a}}_{r}={\hat{a}}_{x}\text{sin}\theta \text{cos}\phi +{\hat{a}}_{y}\text{sin}\theta \text{sin}\phi +{\hat{a}}_{z}\text{cos}\theta$$ (11)

$$\bar{p}={\hat{a}}_{x}x+{\hat{a}}_{y}y+{\hat{a}}_{z}z.$$ (12)

The transformation vector for the spherical coordinates is indicated by ${\hat{a}}_{r}$, and $\bar{p}$ is the position vector of the antenna elements in rectangular coordinates expressed by the unit vectors of ${\hat{a}}_{x}$, ${\hat{a}}_{y}$, and ${\hat{a}}_{z}$.

## Results and analysis

### Antenna characteristics and measurements

**Fig 4:**
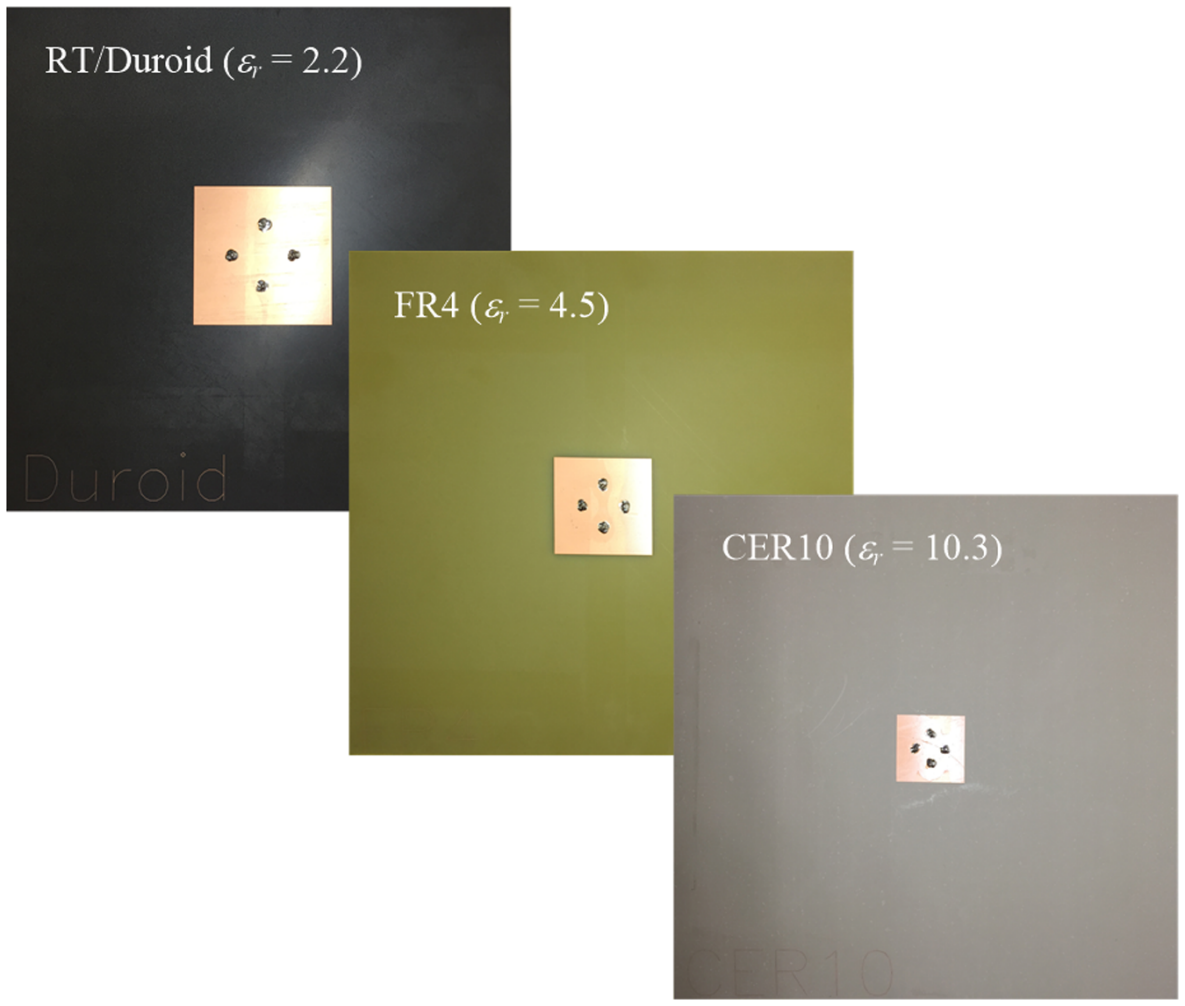
Photograph of the sample SRMP antennas fabricated on RT/Duroid, FR4, and CER10 substrates. The sample SRMP antennas are fabricated on three off-the-shelf substrates to verify variations of aperture size, antenna characteristics, and beamforming performances according to *ε*_*r*_ of the substrates.

One of the major advantages of the SRMP antenna is its smaller aperture size compared to conventional MRMP arrays. To evaluate the SRMP antenna in terms of miniaturization, different substrates of RT/Duroid (*ε*_*r*_ = 2.2, tanδ = 0.0004), FR4 (*ε*_*r*_ = 4.5, tanδ = 0.02), and CER10 (*ε*_*r*_ = 10.3, tanδ = 0.0035) are used to fabricate three sample SRMP antennas, as presented in Fig 4. These substrates with different dielectric constants are used to observe variations of aperture size, antenna characteristics, and beamforming capability through measurements. It is assumed that the antenna has four ports and operates in the L band (1 GHz ≤ *frequency* ≤ 2 GHz). The resonant frequencies of the sample antennas are tuned using full-wave EM simulations, and the design parameters are listed in Table 1. The aperture areas of the fabricated sample antennas are 39.4 cm^2^, 19.9 cm^2^, and 9.3 cm^2^, which are reduced in inverse proportion to *ε*_*r*_, and their antenna characteristics, such as scattering parameters, bore-sight gains, and active element patterns, are measured in a full anechoic chamber.

**Table 1:** Detailed values of the design parameters of the three sample SRMP antennas.

Parameters	RT/Duroid	FR4	CER10
*w*	62.8 mm	44.6 mm	30.5 mm
*d*	14.1 mm	9.4 mm	6.7 mm
*h*	1.6 mm	1.6 mm	1.6 mm

**Fig 5:**
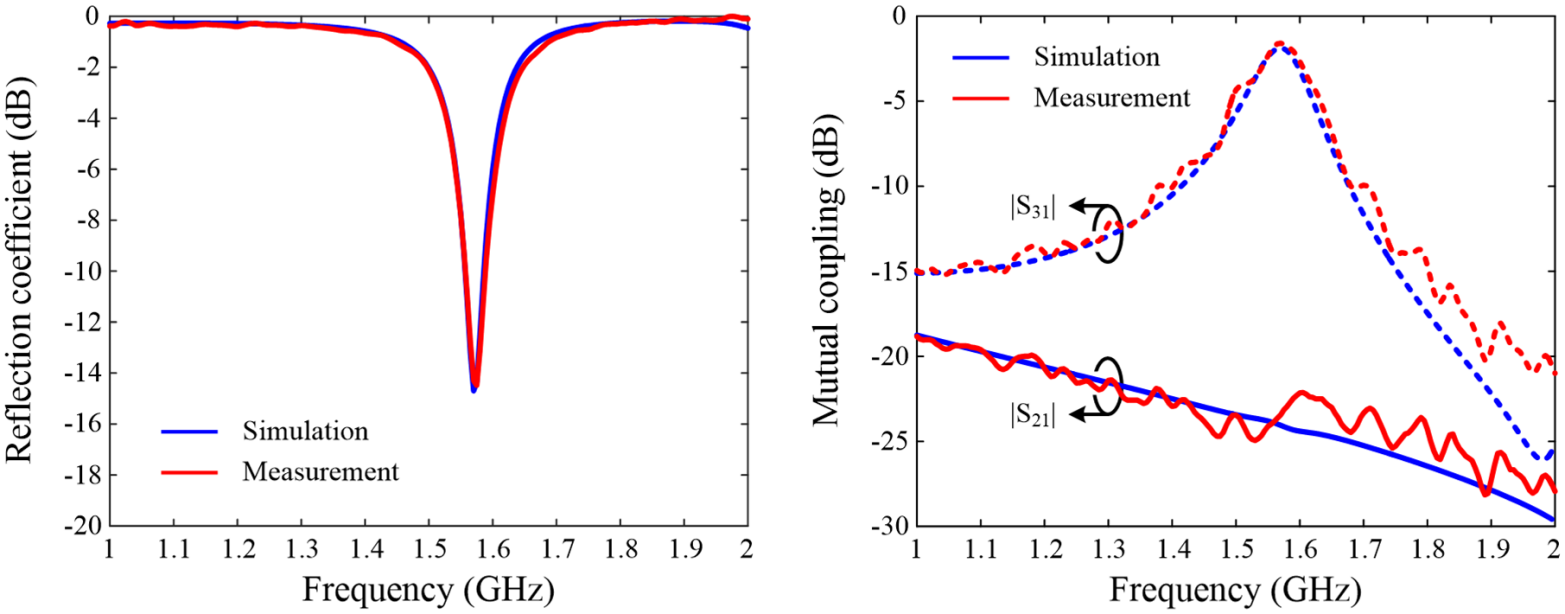
Measured scattering parameters of the sample SRMP antenna fabricated on the RT/Duroid substrate in comparison with simulated results. Simulated and measured results of scattering parameters are presented as a function of frequency. (A) |S_11_|. Blue line indicates the simulated results, and measured data are specified in red. (B) |S_21_| and |S_31_|. Here, solid blue and red lines present the simulated and measured results of the mutual coupling between Port 1 and Port 2, and dotted lines indicate the coupling between Port 1 and Port 3.

Fig 5 shows the measured scattering parameters of the SRMP antenna fabricated on the RT/Duroid substrate as a function of frequency compared to the results obtained from the EM simulation (*see* S2 Fig in the supplementary information for an illustration of the measured results of other fabricated antennas). The antenna is well-matched at 1.575 GHz with |S_11_| values of ‒14.5 dB and ‒14.4 dB for the measurement and the simulation, respectively, and the measured 10-dB matching bandwidth of 32 MHz shows good agreement with the simulated value of 29 MHz. Since the antenna ports are placed in close proximity (20 mm, ≤ 0.1λ_0_ at 1.5 GHz) and share the same radiator, the mutual coupling between Port 1 and Port 3, denoted as |S_31_|, is relatively stronger with a peak value of ‒1.7 dB for measurement and ‒1.9 dB for simulation at 1.575 GHz. However, the coupling strength between Port 1 and Port 2, denoted as |S_21_|, decreases to ‒23.2 dB for measurement and ‒24.1 dB for simulation due to the orthogonal polarization.

### Analysis of coupling effects

**Fig 6:**
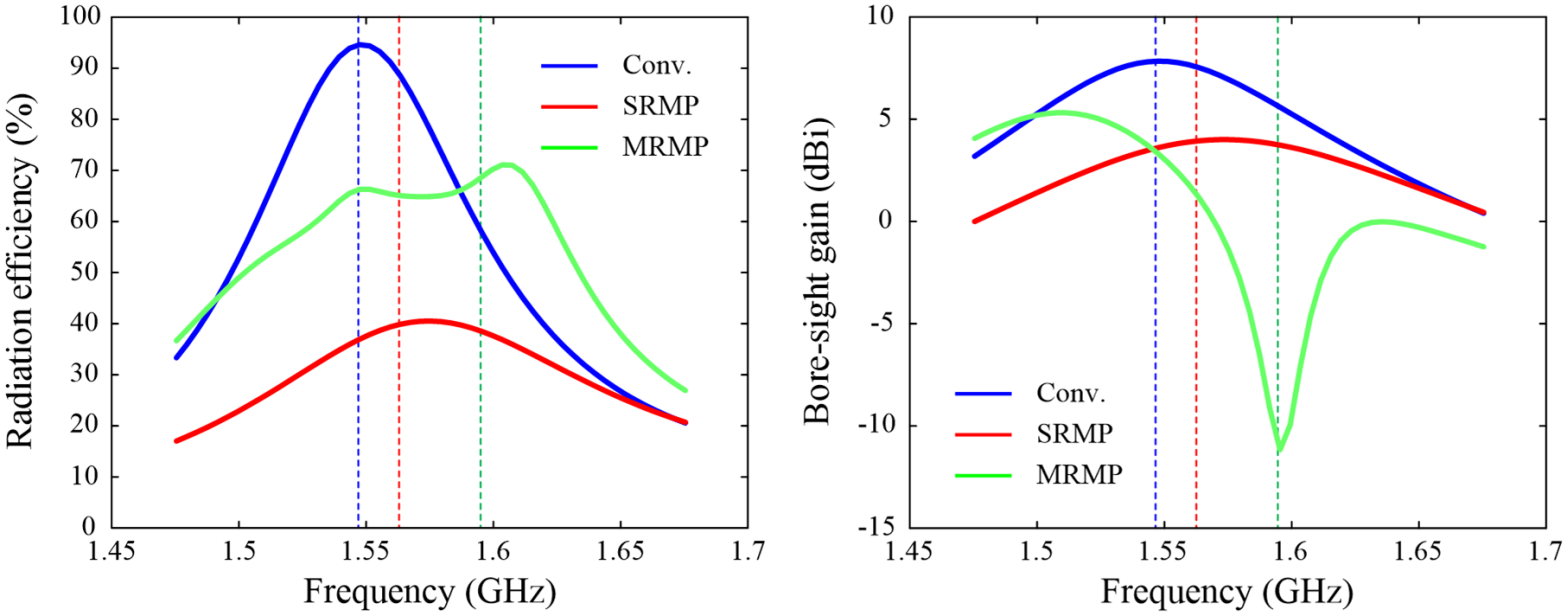
Comparison of the radiation efficiency and bore-sight gain for verifying the coupling effect. The SRMP and MRMP structures used in this comparison have similar aperture sizes of 62.9 × 62.9 mm^2^ and 65.1 × 65.1 mm^2^, respectively, by applying different dielectric constants of *ε*_*r*_ = 2 and *ε*_*r*_ = 8. (A) Active reflection coefficients of the SRMP antenna in comparison with the MRMP array at Port 1. (B) Active element patterns compared to the isolated pattern at Port 1.

To analyze the effect of the strong mutual coupling, especially between Port 1 and Port 3, the radiation properties of the SRMP antenna are calculated using full-wave EM simulations, and the results are compared to those for the conventional square patch antenna with a single feed and the four-element MRMP array. Note that the three structures have a similar aperture size of about 0.3λ × 0.3λ and that their resonant frequencies are 1.547 GHz (conventional patch), 1.563 GHz (SRMP), and 1.595 GHz (MRMP). Fig 6(a) and 6(b) present the results of the radiation efficiency and the bore-sight gain as a function of frequency, and the efficiency decreases from 94.6% (conventional) to 39.9% (SRMP) as a result of connecting multiple ports to the same radiator, which is lower than 68.7% of the MRMP array. However, the active element pattern of the MRMP array in the bore-sight direction exhibits a blind spot with the gain of –11.2 dBi at its resonant frequency, as shown in Fig 6(b), whereas the SRMP antenna maintains a higher gain of 3.9 dBi. This implies that the SRMP antenna has the advantage of maintaining higher gain in the upper hemisphere without serious blind spots, although its radiation efficiency is reduced by the effect of the coupling.

**Fig 7:**
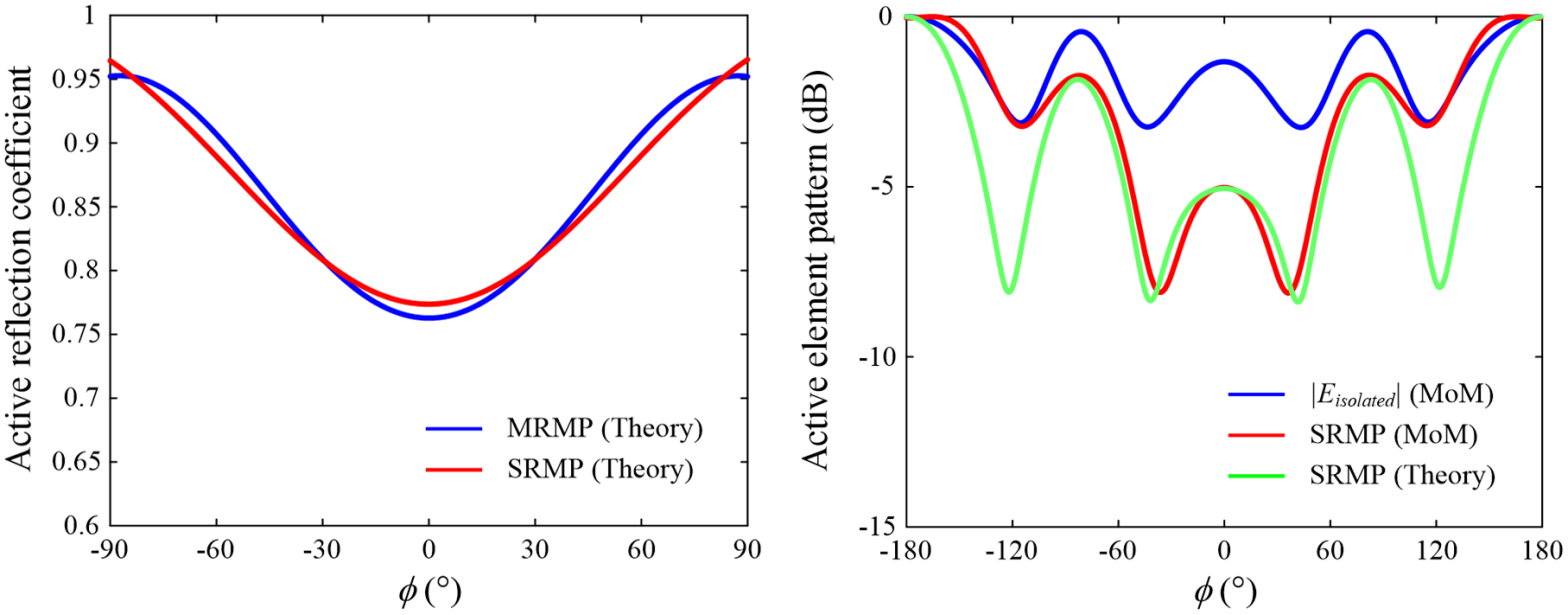
Active reflection coefficients and active element patterns of the SRMP antenna in *ϕ*-direction. The SRMP and MRMP structures used in this comparison have similar aperture sizes of 62.9 × 62.9 mm^2^ and 65.1 × 65.1 mm^2^, respectively, by applying different dielectric constants of *ε*_*r*_ = 2 and *ε*_*r*_ = 8. (A) Active reflection coefficients of the SRMP antenna in comparison with the MRMP array at Port 1. (B) Active element patterns compared to the isolated pattern at Port 1.

Fig 7(a) shows the active reflection coefficient of the SRMP antenna, which is computed by Eq (13), and its values are compared to the results of the MRMP array at Port 1.

$$\Gamma_{m}\left(\phi \right)=\frac{V_{m}^{-}}{V_{m}^{+}}=\frac{\sum_{n=1}^{N}S_{mn}e^{-jU_{n}}}{e^{-jU_{m}}}=\sum_{n=1}^{N}S_{mn}e^{-j\left(U_{n}-U_{m}\right)},$$ (13)

where

$$U_{n}=kd\text{sin}\theta \text{cos}\left(\phi -\phi_{n}\right),$$ (14)

$$U_{m}=kd\text{sin}\theta \text{cos}\left(\phi -\phi_{m}\right).$$ (15)

*m* and *n* indicate indices of antenna ports, and *S*_*mn*_ represents scattering parameters between *m*-th and *n*-th ports. *N* is the number of ports, and *ϕ*_*m*_ and *ϕ*_*n*_ are the angular positions of the corresponding ports. *k* is the wave number, and *d* represents the distance from the center of the SRMP antenna to the port. Due to the strong mutual coupling between ports, the active reflection coefficient increases from 0.77 to 0.96 as |*ϕ*| becomes larger. This degradation also appears in the case of the MRMP array near *ϕ* = ±90°, which implies that the active reflection coefficient of the SRMP antenna is similar to that of the MRMP array when their aperture sizes are almost identical. We also observed active element patterns in comparison with the isolated pattern (|*E*_*isolated*_|), which is calculated using the conventional patch antenna with a single port, as specified by the blue curve in Fig 7(b). The red line is obtained from the full-wave EM simulations using the SRMP antenna (*ε*_*r*_ = 2) at Port 1, and the green line is calculated by the definition shown in Eq (16).

$$E_{m}\left(r,\phi \right)=E_{isolated}\left(r,\phi \right)\left[1+\Gamma_{m}\left(-\phi \right)\right]e^{-jU_{m}},$$ (16)

where Γ_*m*_ represents the active reflection coefficient defined in Eq (13). The results show that the strong coupling lowers the gain of the active element pattern with the maximum deviation of 5.1 dB at *ϕ* = 34°, and this gain reduction can be minimized by improving the isolation properties between ports.

**Fig 8:**
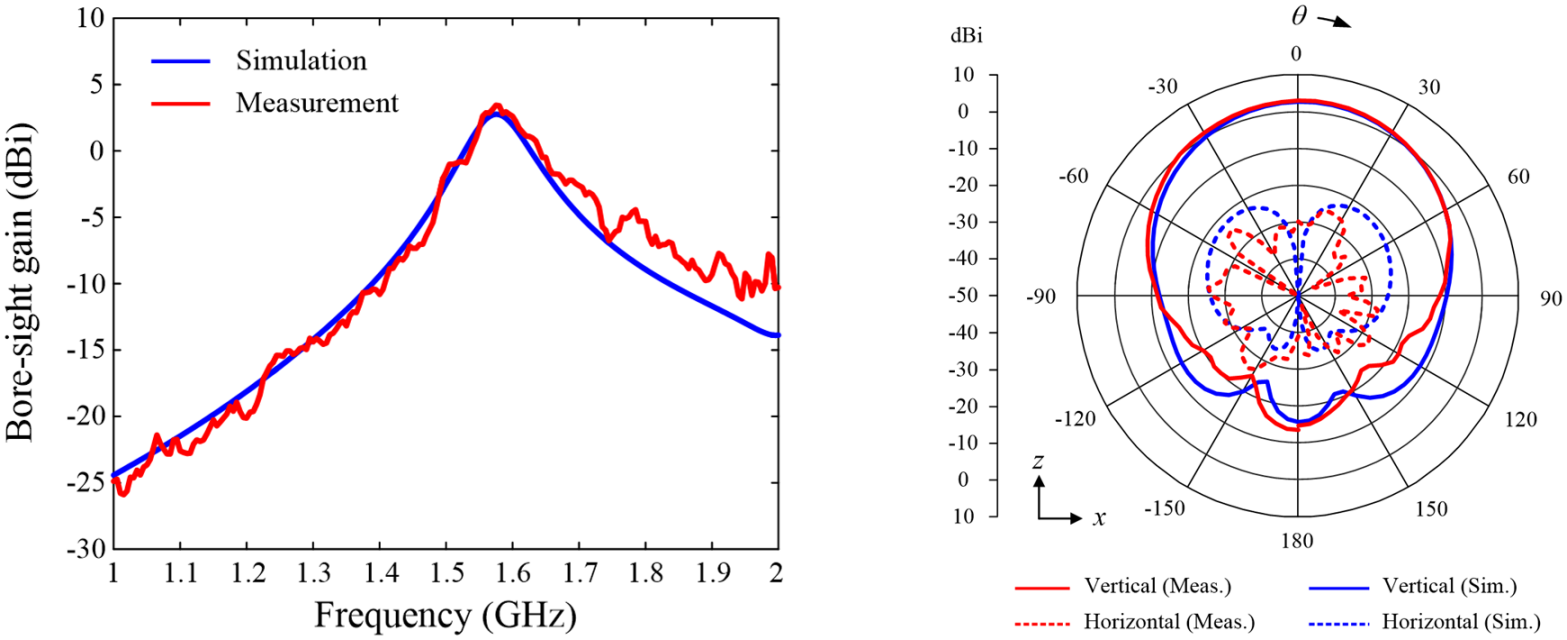
Measured radiation properties of the bore-sight gain and active element patterns at Port 1 in comparison with simulated results. The results presented here are for the sample SRMP antenna fabricated on the RT/Duroid substrate, and the bore-sight gain is obtained from active element patterns at *θ* = 0°. (A) Bore-sight gain. Blue line indicates simulated results, and the red line presents measured data. (B) Active element patterns. Solid and dotted lines present the patterns of the vertical and horizontal polarizations, respectively; red represents results from the measurement, and blue represents results from the simulation.

Fig 8(a) provides a comparison of the measured and simulated bore-sight gains obtained from the active element patterns shown in Fig 8(b). In this comparison, only Port 1 is excited, whereas the other ports are terminated with 50-Ω loads. The measured and simulated gain values in the bore-sight direction at 1.575 GHz are 3.4 dBi and 2.8 dBi, respectively, and the pattern of the antenna at each port is linearly polarized with a cross-polarization level of ‒32.9 dB for measurement and ‒53.3 dB for simulation. The results confirm that the existence of multiple ports connected to the single radiator does not cause serious gain reduction or polarization distortion in the active element patterns (*see* S3 Fig in the supplementary information to verify measured active element patterns of other fabricated antennas).

## Verification and discussion

### Verification of beamforming performance

**Fig 9:**
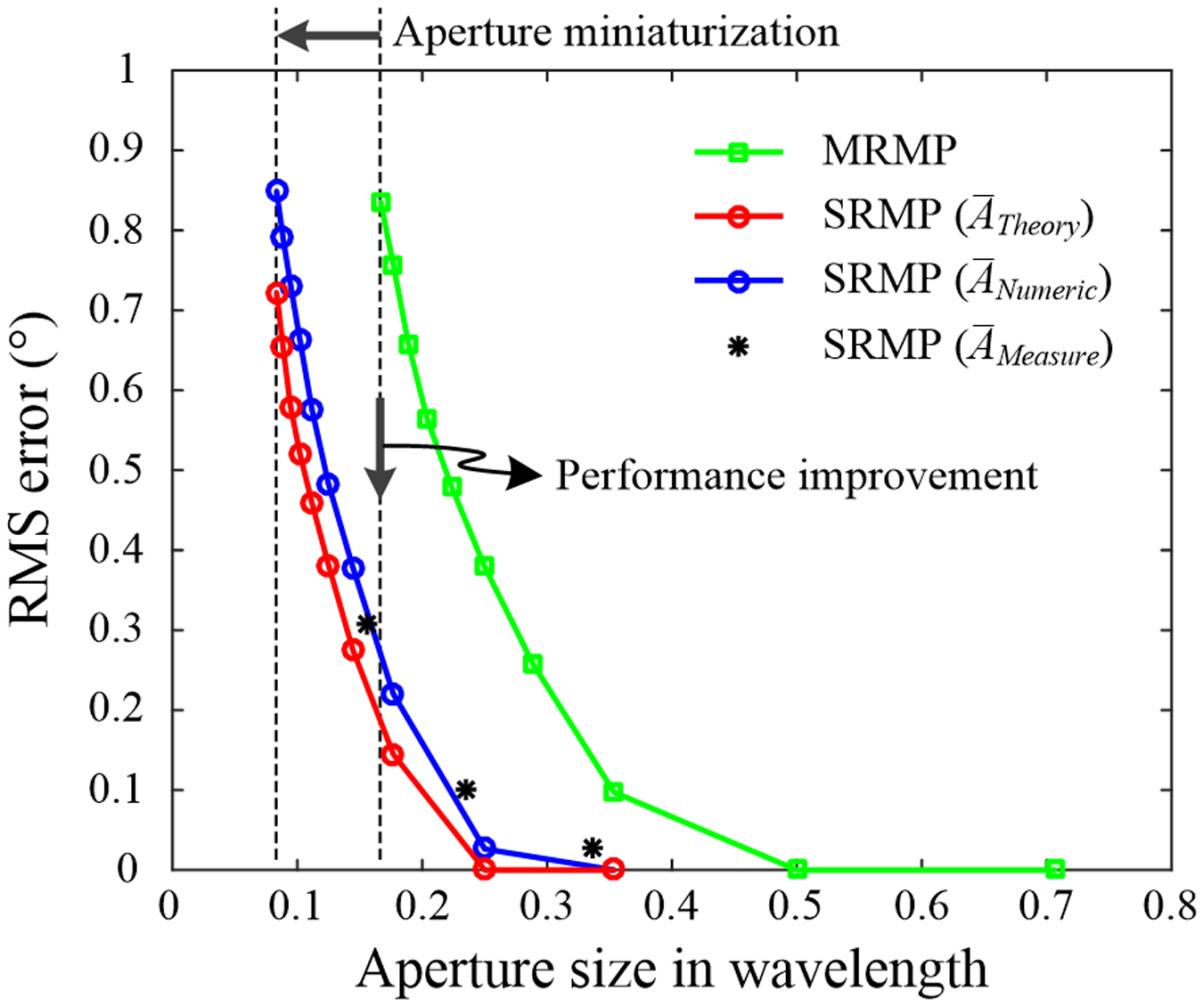
Performance variations of the RMS error for the DoA estimation according to aperture size. The results obtained using ${\bar{A}}_{Theory}$ and ${\bar{A}}_{Numeric}$ are indicated by red and blue lines with circular markers, and the results of ${\bar{A}}_{Measure}$ are specified by black ‘*’ markers. The results of the four-port MRMP array are presented by the green line with square markers.

The beamforming capability of the SRMP antenna is demonstrated in various adaptive beamforming applications, such as the DoA estimation, interference mitigation, and MIMO, through the use of three different approaches: theoretical formulation, numerical full-wave EM simulation [28], and measurement. In the theoretical approach, the formulated array factor *AF*_*SRMP*_ is transformed into a matrix form of the array manifold ${\bar{A}}_{Theory}$, which is composed of steering vectors for arbitrary *θ*- and *ϕ*-directions. The numerical array manifold ${\bar{A}}_{Numeric}$ and the measured array manifold ${\bar{A}}_{Measure}$ are obtained from simulated and measured active element patterns, respectively. Each vector component of these array manifolds is used as an antenna weight to steer array patterns in the adaptive beamforming applications. To examine the fundamental performance limits related to these array manifolds, the aperture sizes of both the SRMP antenna and the MRMP array are scaled by increasing the value of *ε*_*r*_ from 2 to 36, and beamforming performance is evaluated through enumerative studies. Fig 9 shows variations in the root-mean-square (RMS) error of the DoA estimation according to the aperture size in wavelength. Note that the RMS error is defined as the RMS difference between the true and estimated source directions, and the aperture size represents the longitudinal edge length of the square aperture. It is assumed that a single source is placed in the azimuth plane (0° ≤ *ϕ* ≤ 360°, *θ* = 90°), and the incident angle is rotated at intervals of 1°. The direction of the source is then estimated using Bartlett’s beamformer with the assumption that the signal-to-noise ratio is 30 dB [29]. To obtain more reliable results with random noise characteristics, each RMS error is averaged over 100 iterations, and the fundamental performance limits related to ${\bar{A}}_{Theory}$, ${\bar{A}}_{Numeric}$, and ${\bar{A}}_{Measure}$ are calculated as specified by the blue line, the red line, and ‘*’ markers, respectively. The green line shows the results for the four-port MRMP array with a 2 × 2 configuration and is obtained using a numerical array manifold computed by the EM simulation. The fundamental performance limits of the RMS error tend to increase as the aperture size decreases, and the results calculated from the different array manifolds of ${\bar{A}}_{Theory}$, ${\bar{A}}_{Numeric}$, and ${\bar{A}}_{Measure}$ agree well with each other. It is obvious that the SRMP antenna occupies a much smaller space than the MRMP array while maintaining a lower RMS error. For example, the aperture size of the SRMP antenna should be greater than 0.11λ to achieve an RMS error of less than 0.5°; by contrast, to achieve the same RMS error, the minimum aperture size of the MRMP array must be 0.22λ. In addition, the RMS error can be improved from 0.6° to 0.1° by applying the SRMP antenna when the aperture size is limited to less than 0.2λ.

### Field test using the four-channel beamforming hardware

**Fig 10:**
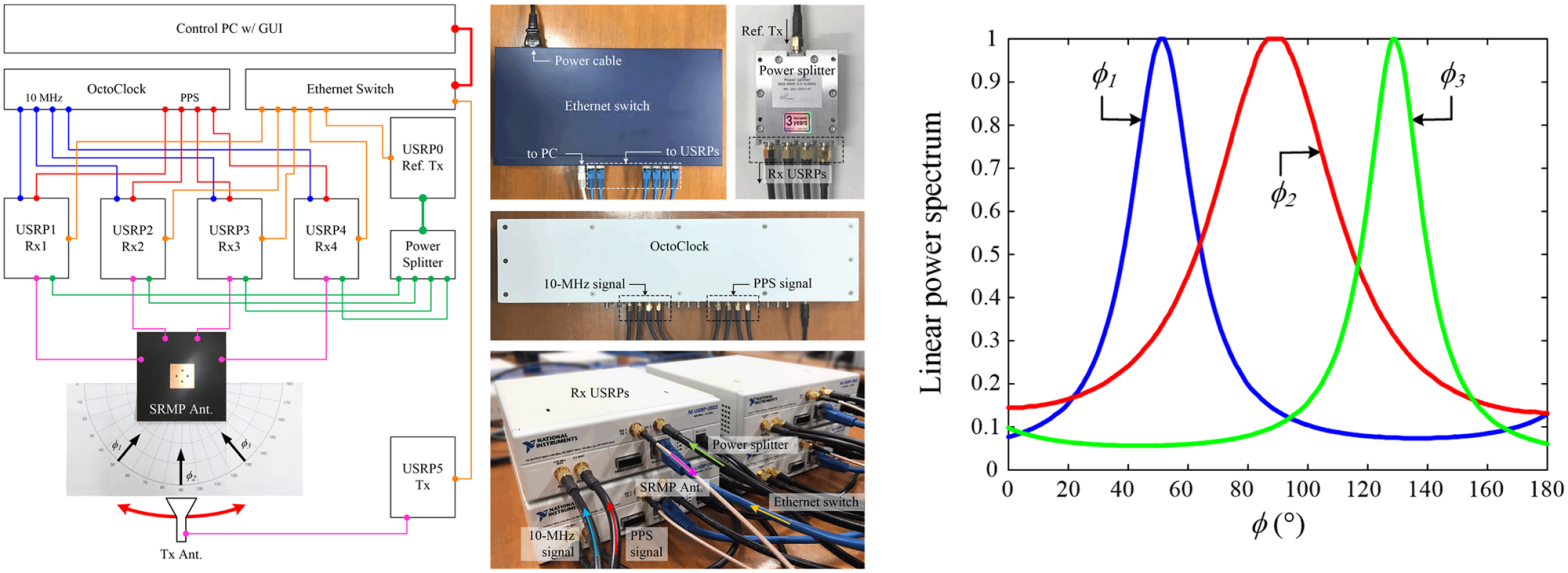
Field tests using the beamforming hardware as the verification of the beamforming capability for the DoA estimation. The port of the SRMP antenna is connected to each receiving channel of the beamforming hardware, and the received signals are used to calculate the covariance matrix for estimating the direction of the source. (A) Configuration of the beamforming hardware. Detailed connections for the beamforming hardware are provided on left, and photographs of the equipment are shown on the right side. (B) Beamforming spectrums of the SRMP antenna. To display the beamforming spectrums, the raw data are imported from the field tests, and the imported spectrums for *ϕ*_*1*_, *ϕ*_*2*_, and *ϕ*_*3*_ are specified by blue, red, and green lines, respectively.

The beamforming capability for the DoA estimation is further validated by field tests using beamforming hardware consisting of six USRPs (Model: NI USRP-2922) from National Instruments, an OctoClock device from Ettus Research, a four-way power splitter from SRTechnology Corp., and an Ethernet switch from NETGEAR. The hardware setup is controlled by LabVIEW Software installed on a computer connected to the Ethernet switch [30], and the results, such as beamforming spectrums and estimated DoAs are visualized with the graphical user interface (GUI) on a display unit. Fig 10(a) shows a hardware configuration with photographs of the beamforming hardware and the SRMP antenna fabricated on the RT/Duroid substrate. The field tests were conducted with respect to three incident angles, that is, *ϕ*_*1*_ = 50°, *ϕ*_*2*_ = 90°, and *ϕ*_*3*_ = 130°, and the beamforming spectrums computed by Bartlett’s beamformer are presented in Fig 10(b). The results demonstrate that the SRMP antenna is capable of steering the spectrum in the direction of interest by adjusting the port weights (*see* S4 Fig in the supplementary information to verify examples of raw data of the time-domain signals obtained from the beamforming hardware and S5 Fig for additional photographs of the field tests with extra GUI results).

**Fig 11:**
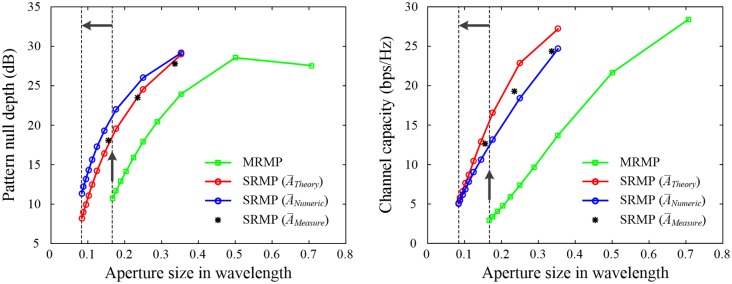
Performance variations of the pattern null depth and the channel capacity in the interference mitigation and MIMO operations. (A) Interference mitigation. (B) MIMO operation. The results obtained using ${\bar{A}}_{Theory}$, ${\bar{A}}_{Numeric}$, and ${\bar{A}}_{Measure}$ are shown by the red line, the blue line, and black ‘*’ markers, respectively, and those of the four-port MRMP array are specified by the green line.

The beamforming application is now extended to the interference mitigation and the MIMO, as shown in Fig 11. In the interference mitigation, the power inversion algorithm is adopted to determine an array pattern with a null steered toward the direction of interference [31], and the depth of the pattern null is used as a figure of merit to evaluate the capability of the interference mitigation [32]. The SRMP antenna can mitigate the power of inference sources with the pattern null depth of 23.3 dB when its aperture size is 0.2λ. The MRMP array, however, requires the larger aperture size of 0.34λ to achieve the same null depth. In addition, a higher relative permittivity should be applied for the MRMP array to maintain the same aperture size. A similar trend is observed for the Ergodic channel capacity in the MIMO operation [33]. The channel capacity is computed using the envelop correlation coefficient of active element patterns [34], and each data point is averaged over 1,000 iterations. The channel capacity of the MRMP array decreases to 4.6 bps/Hz as the aperture size is reduced to 0.2λ, whereas the capacity can increase to 14.8 bps/Hz when applying the SRMP antenna in the same aperture area. The results demonstrate that the SRMP antenna is suitable for miniaturizing aperture size with improved beamforming performances in adaptive beamforming applications.

## Conclusions

We have introduced a new perspective on the SRMP antenna as an innovative approach to the miniaturization of aperture size with improved beamforming performance in an extremely small aperture area. The radiation characteristics of the SRMP antenna were theoretically formulated using the cavity model, and the array factor was electromagnetically characterized for use as complex weights in the DoA estimation, interference mitigation, and the MIMO operation. The fundamental performance limits with respect to aperture size were also investigated using the enumerative studies, and the results were compared to those of the traditional MRMP arrays. For more rigorous demonstration, three sample SRMP antennas were fabricated on the RT/Duroid, FR4, and CER10 substrates, and their beamforming performance was verified by field tests using beamforming hardware. The results proved that the aperture size of the fabricated SRMP antenna could be twice as small as that of the MRMP arrays, and the RMS error, pattern null depth, and channel capacity could also be improved by more than 0.5°, 9.5 dB, and 10.3 bps/Hz, respectively, when the aperture size was restricted to less than 0.2λ. It should be emphasized that the new perspective on the SRMP antenna formulated and demonstrated in this paper provide an innovative solution to the challenge of improving beamforming performance in extremely restricted aperture areas in order to conduct more sophisticated beamforming applications without increasing the number of antennas.

## Supporting information

S1 Fig Variation of the Far-zone fields at Port 1 according to the relative permittivity of the substrate used for the proposed SRMP antenna. (PDF)

S2 Fig Measured reflection coefficients of the three sample SRMP antennas. (PDF)

S3 Fig Measured active element patterns of the three sample SRMP antennas at Port 1. (PDF)

S4 Fig Example raw data of the time-domain signals obtained from the beamforming hardware using the four-port SRMP antenna fabricated on the RT/Duroid substrate. (PDF)

S5 Fig Additional photographs of the field tests with the test environment and the GUI results. (PDF)

## References

[1] Pozar DM. A relation between the active input impedance and the active element pattern of a phased array. IEEE Trans. Antennas Propag. 2003; 51: 2486–2489.

[2] Pozar DM. The active element pattern. IEEE Trans. Antennas and Propag. 1994; 42: 1176–1178.

[3] Wang Y, Du Z. Dual-polarized slot-coupled microstrip antenna array with stable active element pattern. IEEE Trans. Antennas Propag. 2015; 63: 4239–4244.

[4] He QQ, Wang BZ, Shao W. Radiation pattern calculation for arbitrary conformal arrays that include mutual-coupling effects. IEEE Antennas Propag. Mag. 2010; 52: 57–63.

[5] Rubio J, Izquierdo JF, Córcoles J. Mutual coupling compensation matrices for transmitting and receiving arrays. IEEE Trans. Antennas Propag. 2015; 63: 839–843.

[6] Daniel JP. Mutual coupling between antennas for emission or reception—Application to passive and active dipoles. IEEE Trans. Antennas Propag. 1974; 22: 347–349.

[7] Niow CH, Yu YT, Hui HT. Compensate for the coupled radiation patterns of compact transmitting antenna arrays. IET Microw. Antennas Propag. 2011; 5: 699–704.

[8] Kerkho A, Ling H. A simplified method for reducing mutual coupling effects in low frequency radio telescope phased arrays. IEEE Trans. Antennas Propag. 2011; 59: 1838–1845.

[9] Byun G, Choo H, Kim S. Array configuration optimisation of dual-band controlled reception pattern antenna arrays for anisotropic ground platforms. IET Microw. Antennas Propag. 2014; 8: 597–603.

[10] Hui HT. Improved compensation for the mutual coupling effect in a dipole array for direction finding. IEEE Trans. Antennas Propag. 2003; 51: 2498–2503.

[11] Gupta IJ, Ksienski AA. Effect of mutual coupling on the performance of adaptive arrays. IEEE Trans. Antennas Propag. 1983; AP-31: 785–791.

[12] Svendsen ASC, Gupta I J. The effect of mutual coupling on the nulling performance of adaptive antennas. IEEE Antennas Propag. Mag. 2012; 54: 17–38.

[13] Friel EM, Pasala KM. Effects of mutual coupling on the performance of STAP antenna arrays. IEEE Trans. Aerosp. Electron. Syst. 2000; 36: 518–527.

[14] Byun G, Choo H, Ling H. Optimum placement of DF antenna elements for accurate DOA estimation in a harsh platform environment. IEEE Trans. Antennas Propag. 2013; 61: 4783–4791.

[15] Byun G, Choo H, Kim S. Design of a small arc-shaped antenna array with high isolation for applications of controlled reception pattern antennas. IEEE Trans. Antennas Propag. 2016; 64: 1542–1546.

[16] Lu S, Hui HT, Bialkowski M. Optimizing MIMO channel capacities under the influence of antenna mutual coupling. IEEE Antennas Wirel. Propag. Lett. 2008; 7: 287–290.

[17] Lu JH, Wong KL. Slot-loaded meandered rectangular microstrip antenna with compact dual frequency operation. Electron. Lett. 1998; 34: 1048–1050.

[18] Azaro R, Boato G, Donelli M, Franceschini G, Martini A, Massa A. Design of miniaturised ISM-band fractal antenna. Electron. Lett. 2005; 41: 785–786.

[19] Gupta S, Muncu G. Dual-band miniature coupled double loop GPS antenna loaded with lumped capacitors and inductive pins. IEEE Trans. Antennas Propag. 2013; 61: 2904–2910.

[20] Choo H, Rogers RL, Ling H. Design of electrically small wire antennas using a Pareto genetic algorithm. IEEE Trans. Antennas Propag. 2005; 53: 1038–1046.

[21] Bowers SM, Hajimiri A. Multi-port driven radiators. IEEE Trans. Microw. Theory Tech. 2013; 61: 4428–4441.

[22] Wang R, Wang BZ, Gong ZS, Ding X. Compact multiport antenna with radiator-sharing approach and its performance evaluation of time reversal in an intra-car environment. IEEE Trans. Antennas Propag. 2015; 63: 4213–4219.

[23] Balanis CA. Antenna Theory: Analysis and Design. 3rd ed New York: Wiley; 2005 pp. 826–839.

[24] National Instruments Corporation. USRP Model: NI USRP-2922. 2016. http://www.ni.com.

[25] Ettus Research. OctoClock Model: OctoClock CDA-2990. 2016. http://www.ettus.com.

[26] SRTechnology Corporation. Power Splitter Model: SMA 4-WAY 0.5 ~ 6 GHz. 2016. http://www.srt.kr.

[27] NETGEAR. Ethernet Switch Model: NETGEAR ProSAFE 16 Port Gigabit Switch. 2016. http://netgear.com.

[28] Altair Engineering. FEKO Suite 7.0. 2016. http://www.feko.info.

[29] Krim H, Viberg M. Two decades of array signal processing research: the parametric approach. IEEE Signal Process. Mag. 1996; 13: 67–94.

[30] National Instruments Corporation. LabVIEW System Design Software. 2016. http://www.ni.com.

[31] Compton RT. The power-inversion adaptive array: concept and performance. IEEE Trans. Aerosp. Electron. Syst. 1979; AES-15: 803–814.

[32] Byun G, Choo H, Kim S. Improvement of pattern null depth and width using a curved array with two subarrays for CRPA systems. IEEE Trans. Antennas Propag. 2015; 63: 2824–2827.

[33] Foschini GJ, Gans MJ. On limits of wireless communications in a fading environment when using multiple antennas. Wireless Pers. Commun. 1998; 6: 311–335.

[34] Dong L, Choo H, Heath RW, Ling G. Simulation of MIMO channel capacity with antenna polarization diversity. IEEE Trans. Wirel Commun. 2005; 4: 1869–1873.

