# Supplementary material for: New perspective on single-radiator multiple-port antennas for adaptive beamforming applications
Source: PLoS One. 2017 Oct 12;12(10):e0186099. doi: 10.1371/journal.pone.0186099 (PMC5638333; doi:10.1371/journal.pone.0186099)
Supplement: S1 Fig — (PDF) [file pone.0186099.s001.pdf]

S1 Fig

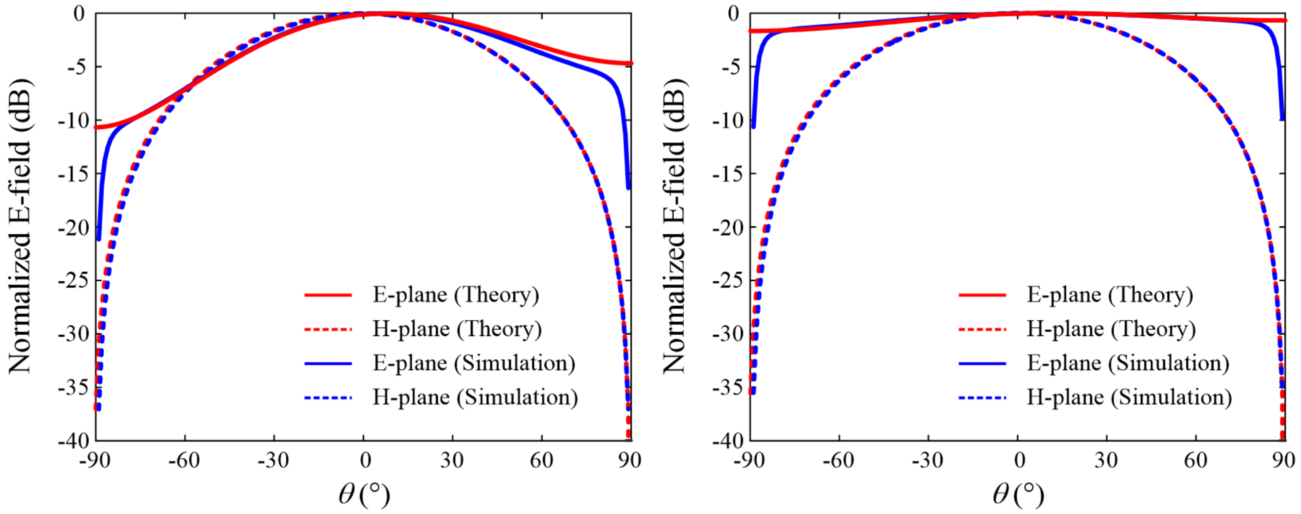

(A) (B)

S1 Fig. Variation of the Far-zone fields at Port 1 according to the relative permittivity of the substrate used for the proposed SRMP antenna.

S1 Fig shows a comparison of the electric fields in the E- and H-planes expressed by solid and dotted lines, respectively. The results, that are calculated using the equation (1) of the manuscript, are specified in red, and the data obtained using the full-wave EM simulation are indicated by blue lines. Due to the phase delay β_d, the E-plane field is steered toward the positive θ-direction for both results, and the degree of the steered angle tends to decrease as the value of the relative permittivity ε_r is increased from 2 to 10. For example, the deviation between the gain value of $\theta = +90°$ and that of $\theta = -90°$ is 6 dB in case of $\varepsilon_r = 2$ and decreases to 1 dB for an increased ε_r value of 10. Note that the deviation of $\varepsilon_r = 4$, presented in Fig 3 of the manuscript, is 2.9 dB, which implies that the steered angle gradually decreases in accordance with the ε_r value that changes the absolute value of β_d. (A) Normalized E-field when $\varepsilon_r = 2$. (B) Normalized E-field when $\varepsilon_r = 10$.
