# Supplementary material for: New perspective on single-radiator multiple-port antennas for adaptive beamforming applications
Source: PLoS One. 2017 Oct 12;12(10):e0186099. doi: 10.1371/journal.pone.0186099 (PMC5638333; doi:10.1371/journal.pone.0186099)
Supplement: S2 Fig — (PDF) [file pone.0186099.s002.pdf]

S2 Fig

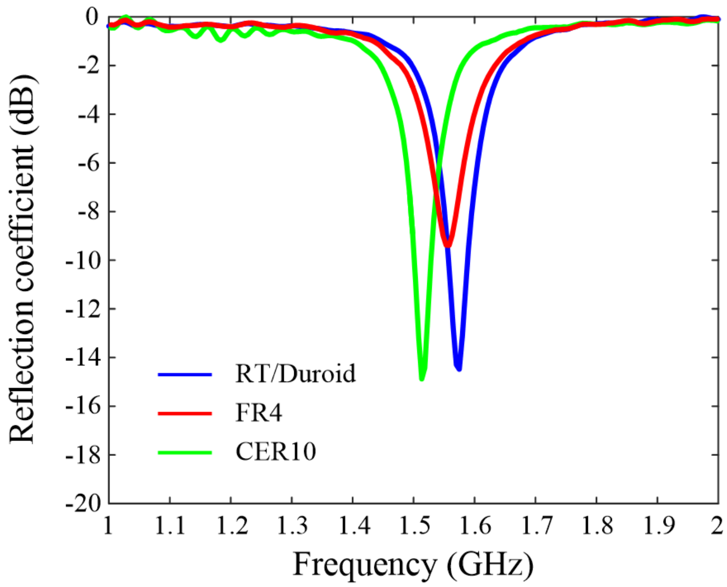

S2 Fig. Measured reflection coefficients of the three sample SRMP antennas.

S2 Fig provides measured reflection coefficients at Port 1 as a function of frequency. The antennas are well-matched with the minimum values of –14.5 dB, –9.4 dB, and –14.9 dB, and their resonant frequencies are 1.575 GHz, 1.555 GHz, and 1.51 GHz for RT/Duroid (blue line), FR4 (red line), and CER10 (green line) substrates, respectively. The minimum value of the FR4 substrate is higher than the others because its quality factor is relatively lower due to a higher loss tangent of 0.02. Note that the loss tangents of the RT/Duroid and the CER10 substrates are 0.0004 and 0.0035, respectively.
