# Supplementary material for: New perspective on single-radiator multiple-port antennas for adaptive beamforming applications
Source: PLoS One. 2017 Oct 12;12(10):e0186099. doi: 10.1371/journal.pone.0186099 (PMC5638333; doi:10.1371/journal.pone.0186099)
Supplement: S3 Fig — (PDF) [file pone.0186099.s003.pdf]

S3 Fig

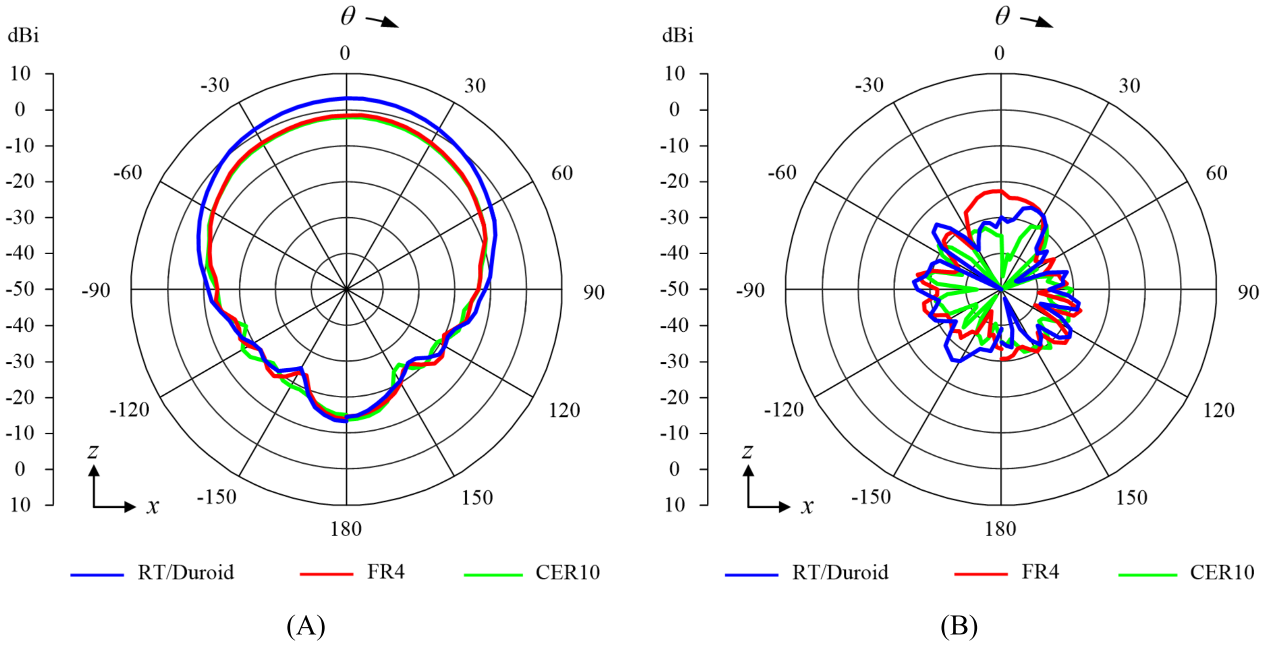

S3 Fig. Measured active element patterns of the three sample SRMP antennas at Port 1.

S3 Fig presents a comparison of measured active element patterns for vertical and horizontal polarizations. It can be seen that the three sample antennas are linearly polarized at each port, and their cross-polarization levels are –32.9 dB (RT/Duroid), –20.9 dB (FR4), and –32.9 dB (CER10) in the bore-sight direction. The measured bore-sight gains are 3.1 dBi (RT/Duroid), –1.7 dBi (FR4), and –2.2 dBi (CER10), and the gain values of the FR4 substrate are similar to that of the CER10 substrate due to the low quality factor discussed in S2 Fig. We can also verify that the existence of multiple ports sharing a single radiator does not cause any serious pattern distortion in the upper hemisphere. (A) Vertical polarization. (B) Horizontal polarization.
