# Supplementary material for: New perspective on single-radiator multiple-port antennas for adaptive beamforming applications
Source: PLoS One. 2017 Oct 12;12(10):e0186099. doi: 10.1371/journal.pone.0186099 (PMC5638333; doi:10.1371/journal.pone.0186099)
Supplement: S4 Fig — (PDF) [file pone.0186099.s004.pdf]

S4 Fig

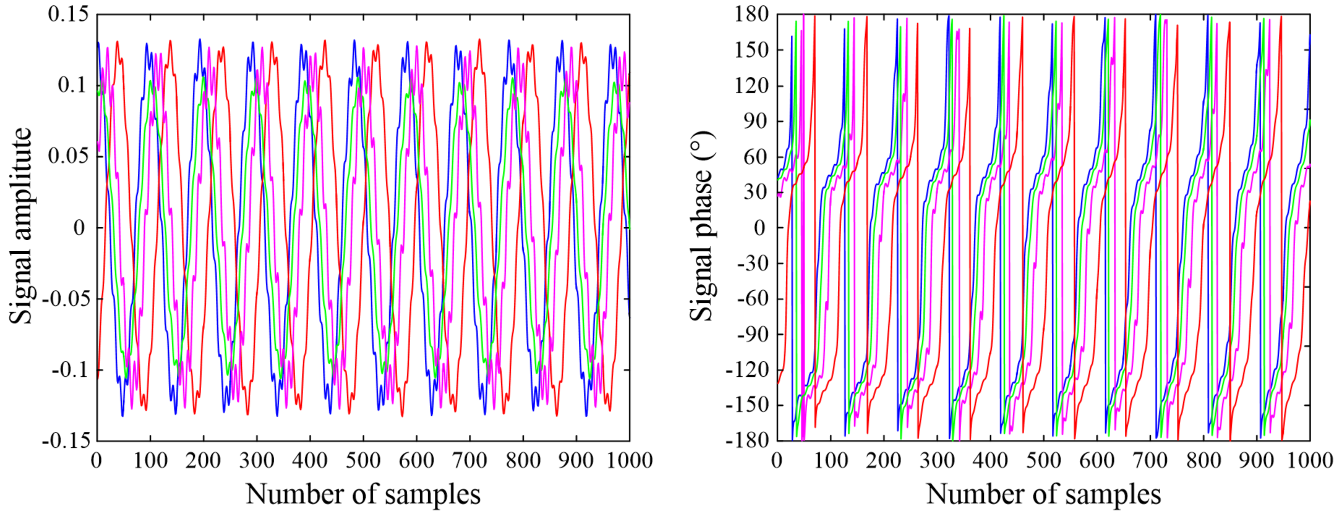

(A) (B)

S4 Fig. Example raw data of the time-domain signals obtained from the beamforming hardware using the four-port SRMP antenna fabricated on the RT/Duroid substrate.

S4 Fig shows the amplitude and phase variations of time-domain signals received by four ports of the sample SRMP antenna fabricated on the RT/Duroid substrate when a source is located at $\phi_l = 50°$: the signals incident to Port 1, Port 2, Port3, and Port 4 are specified by blue, red, green, and magenta solid lines, respectively. The complex time-domain signal is obtained from the I/Q channels of the beamforming hardware composed of six USRPs, and the signal received by each port is weighted-and-summed to form desired active array patterns. As discussed in the manuscript, the time delay and the amplitude difference between ports vary in accordance with the source direction, therefore, we think that the proposed SRMP antenna can be extended to more sophisticated beamforming applications that require extremely miniaturized front-end components. (A) Amplitude variations of the received signal. (B) Phase variations of the received signal.
