# Supplementary material for: New perspective on single-radiator multiple-port antennas for adaptive beamforming applications
Source: PLoS One. 2017 Oct 12;12(10):e0186099. doi: 10.1371/journal.pone.0186099 (PMC5638333; doi:10.1371/journal.pone.0186099)
Supplement: S5 Fig — (PDF) [file pone.0186099.s005.pdf]

S5 Fig

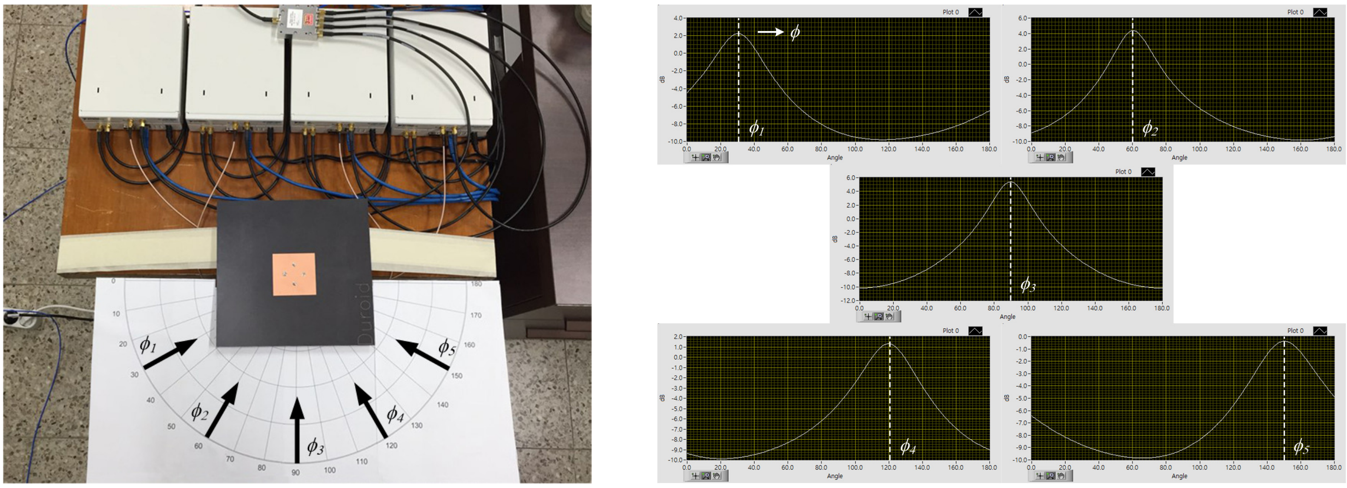

(A) (B)

S5 Fig. Additional photographs of the field tests with the test environment and the GUI results.

S5 Fig presents photographs of the test environment using the beamforming hardware and the GUI spectrums taken during the field tests with the sample SRMP antenna printed on the RT/Duroid substrate. The field tests were conducted for a source located in the azimuth direction ($0° \leq \phi \leq 360°$, $\theta = 90°$), and the beamforming spectrums displayed on the GUI were captured for five incident angles: $\phi_1 = 30°$, $\phi_2 = 60°$, $\phi_3 = 90°$, $\phi_4 = 120°$, and $\phi_5 = 150°$. As can be seen, the peak direction of each beamforming spectrum matches well with the true source direction that is specified by a white dashed line in the GUI results. (A) Test environment with the beamforming hardware. (B) Photographs of the GUI spectrums taken during the field tests.
